# The influence of family function on online prosocial behaviors of high school students: A moderated chained mediation model

**DOI:** 10.3389/fpsyg.2023.1103897

**Published:** 2023-03-01

**Authors:** Lulu Cui, Zhaoliang Li

**Affiliations:** Department of Psychology, School of Philosophy and Sociology, Jilin University, Changchun, China

**Keywords:** family function, online prosocial behaviors, empathy, social support, high school students

## Abstract

The frequency of cyberbullying incidents is gradually increasing, and the seriousness of the consequences is gradually becoming more prominent. Previous studies have shown that cyberbullying bystander behaviors play an important role in reducing cyberbullying. This study aims to explore the mechanisms that high school students’ family function, empathy, and social support levels how to affect their implementation of online prosocial behaviors when they act as cyberbullying bystanders. The study was conducted in 1961 high school students (*M* = 16.84 years; SD = 1.08) in China. Results found that family function promotes online prosocial behaviors through (a) empathy, (b) social support, and (c) chain mediating path of empathy and social support. There were interactions between gender and family function as well as social support, which played a moderating role in the paths of family function and online prosocial behaviors and social support and online prosocial behaviors, respectively. We investigated how family function affected online prosocial behaviors in high school students and how empathy and social support worked to promote them to carry out online prosocial behaviors.

## Introduction

According to the 50th China Internet Development Statistical Report released by the China Internet Network Information Center in 2022, the number of netizens in China reached 1.051 billion until June 2022, and the Internet penetration rate reached 74.4%, of which the scale of netizens aged 10–19 reached 142 million, accounting for 13.5% of the total netizens (China Internet Network Information Center, 2022). It could be concluded from the data of the report that the Internet occupied a high penetration rate in people’s life, and some behavioral tendencies of people could be reflected through the network social platforms (Chen et al., 2022). With the further expansion of the scale of netizens in China and the further increase in the proportion of adolescents and minors, cyberbullying has gradually become the focus of scholars and researchers. Cyberbullying refers to the use of electronic media (such as social networks, email, chat, text, MMS, etc.) in an online environment to do harm to someone who is unable to defend themselves. It is intentional aggressive behavior that demonstrates strength or competence characteristics of imbalance (Palermiti et al., 2017). In a study of bullying behavior among 7,182 middle and high school students in the United States, 8.3% of students reported that they had committed cyberbullying, 9.8% of them reported that they had been subjected to cyberbullying, and 13.6% of students reported had been both the perpetrator and the bullied in cyberbullying in the past 2 months (Wang et al., 2009). Many researchers at home and abroad have shown through empirical researches that cyberbullying had a significant negative impact on the victim’s campus environment, physical and mental health, interpersonal communication, and emotions (González-Cabrera et al., 2018). As a result, both the cyberbullies and the perpetrators showed higher tendencies to depression and loneliness (Varghese and Pistole, 2017), greater insecurity and attachment behaviors, lower levels of self-esteem (Varghese and Pistole, 2017; González-Cabrera et al., 2018), a higher level of social anxiety (Pabian and Vandebosch, 2015), higher suicidal tendencies (Mitchell et al., 2016) and more alcohol abuse behaviors (Selkie et al., 2015).

### Online prosocial behavior

There are three important roles in both traditional bullying and cyberbullying incidents, that is, cyberbullying perpetrators, cyberbullies, and cyberbullying bystanders (Li, 2010). Among them, bystanders refer to individuals who are at present but do not participate in events or situations, and their synonymous expressions are passers-by, witnesses, etc. (Stueve et al., 2006). In a study of Nichatolas Brody, it was pointed out that among the three roles in cyberbullying, researchers paid more attention to the motivation and implementation of the perpetrator and the bullied, while the research papers from the perspective of bystanders were relatively in a small quantity (Brody and Vangelisti, 2015). Foreign researchers have divided the responses of bystanders to cyberbullying into two modes, namely the positive mode of protecting the bullied and the negative mode of helping the bullying (Olenik-Shemesh et al., 2016). Domestic researchers believed that when individuals faced cyberbullying incidents and acted the role of bystanders, their responses could be roughly divided into three categories, specifically behaviors that promote bullying, behaviors that protect the bullied, and outsider behaviors (Teng, 2015). In cyberbullying incidents, the reactions of protecting the victim were seen as a cyber-prosocial behavior or online prosocial behavior, which played a significant role in the intervention of cyberbullying incidents, and it might affect the frequency of bullying incidents and the developmental outcomes of incidents (Salmivalli et al., 2011). Online prosocial behaviors refer to behaviors that are beneficial to others and the society in online context, and are not based on receiving rewards as the aim of these behaviors (Jiang et al., 2017). In a study of Eisenberg, there was no clear definition and conceptual distinction between altruistic behaviors and prosocial behaviors, so this study did not distinguish between online altruistic behaviors and online prosocial behaviors as well (Eisenberg et al., 2002). Applying naturalistic observations research methods on school playgrounds investigations, researchers studied peer intervention for bullying and found that in two-thirds of cases, peer intervention was effective in stopping bullying within 10 s and interventions in preventing bullying were equally effective both in boys and girls (Hawkins et al., 2001). Some researchers also used the method of constructing a multi-level model for 6,764 participants and came to the conclusion that with the face of bullying incidents, bystanders adopted prosocial behaviors to protect the bullied could effectively reduce the frequency of bullying (Salmivalli et al., 2011). Moreover, protecting the bullied person could also effectively reduce the victims’ feelings of stress, anxiety, and depression, and allowed the victim to receive positive feedback (Salmivalli, 2010).

Guided by the General Learning Model, the researchers conducted a series of studies on the impact of the bystander effect on individual behaviors, and the results showed that exposure to online violent games and violent videos could obviously increase the probability of cyberbullying behaviors as well as decrease the probability of online prosocial behaviors (Bartholow and Anderson, 2002), however, watching prosocial videos or listening to prosocial music could increase individual’s prosocial behaviors (Fireman et al., 2010). Some researchers adopted experimental research methods to study the bystander effect in online prosocial behaviors. By comparing the differences between mass sending helping emails online and sending helping emails with a single name on the Internet, the researchers found that the response rate of sending emails for help individually was much higher than mass sending, and non-bulk helping letters received longer replies, more relevant contents and higher intention for help (Barron and Yechiam, 2002; Blair et al., 2010). This could also indicate that with the face of cyberbullying incidents or online help-seeking incidents, whether there were bystanders or not had a significant impact on individual’s implementation of online prosocial behaviors. Therefore, it was very valuable to study online prosocial behaviors from the perspective of cyberbullying bystanders when people were faced with cyberbullying accidents.

### Family function

Family function refers to the effectiveness of various activities between family members, including family communication, family emotional connection, family internal rules and dealing with external things, and so on (Olson et al., 1979). Olson divided family functions into two dimensions, that is, family cohesion and family adaptability. Family cohesion refers to the emotional bonds between family members and the standards of personal autonomy of a family member in their family system. Family adaptability refers to the ability of the family system to change its power structure, role relationships, and corresponding rules when dealing with external situations and developmental pressures. And according to the Circumplex Model of family system which was developed by Olson, 16 kinds of marital and family systems were divided and identified (Olson et al., 1979). In a later study by Olson, family functions were further divided into three dimensions: family cohesion, family flexibility, and family communication skills (Olson, 2000). Beavers believed that family function included two dimensions, the family’s ability to cope with stress and the style of family interaction (Beavers and Hampson, 2000). Mcmaster’s Family Functional Model Theory claimed that the structure and organization of the family were important factors that strongly influenced and even determined the behaviors of family members (Miller et al., 2000). Studies have shown that family function had a significant impact on depression, suicidal tendencies, and so on, with the result that the better the family function was, the less behavioral and psychological problems individuals had (Keitner and Miller, 1990). Family function was not only closely related to the individual’s emotional response ability and emotional involvement ability, but also significantly related to the control of personal behaviors. Individuals with better family function were more likely to carry out prosocial behaviors (Miller et al., 2000). Some studies have found that the quality of parent–child relationship had a significant impact on the impulsivity of adolescents’ bad behaviors as well as their altruistic behaviors (Lu et al., 1998). Other studies have shown that adolescents’ socialization willingness and socialization results were important reflections of the socialization process of people’s parenting styles and parenting practices, and prosocial behaviors were important parts of adolescent socialization (Darling and Steinberg, 1993), so family functions had a significant impact on online prosocial behaviors. Therefore, the first hypothesis of this study is put forward, H1: family function will positively predict individual’s online prosocial behaviors, and people who have better family function will deliver more online prosocial behaviors.

### Empathy

Empathy, as a critical capacity in our emotional and social lives, is conceptualized as the ability to share the feelings of others (Bernhardt and Singer, 2012). In two other studies by Singer, it was believed that when an individual observed or imagined the emotional state of another person, the observer would develop the state of empathy (Singer and Lamm, 2009; Bernhardt and Singer, 2012). Many researchers held that empathy consists of two components, cognitive empathy and affective empathy (Gladstein, 1983). Davis divided empathy into four dimensions, namely perspective taking (PT), fantasy (FS), empathy concern (EC), and personal distress (PD) (Davis, 1983). In a longitudinal study of 180 children, the results showed that parental emotional warmth and positive expressions could significantly promote children’s empathy-related responses and their social functions (Zhou et al., 2002). In another longitudinal survey of 678 high school students of Belgian nationality, the results demonstrated that to a certain extent, students who grew with better family function were more likely to develop better empathy ability (Miklikowska et al., 2011), which provided evidence for the impact of perceived supportive parenting during adolescence on the development of empathy. In a study of adolescents aged 13 to 18, empathy significantly predicted prosocial behaviors (Silke et al., 2018). And in a study on the role of empathy in improving inter-group relations, the results revealed that empathy could enhance inter-group relations and promote individual’s prosocial behaviors (Stephan and Finlay, 1999). According to previous empirical researches and logical reasoning, the second hypothesis of this study is proposed, H2: Empathy plays a mediating role in the influence of family function on online prosocial behaviors, that is family function can indirectly influence the implementation of online prosocial behaviors through the mediating effect of empathy.

### Social support

Social support refers to information that leads individuals to believe that they are cared for, loved, and respected, additionally, each of them is a part of a team (Cobb, 1976). The concept also aims to draw attention to and focus on resources that may amortize or attenuate the impact of life events and other pressure sources (Coyne and Downey, 1991). In a study of 863 Australian suburban residents by Gavin, social support was divided into three dimensions, that is, support in crisis situations, interaction between neighbors, and community participation (Andrews et al., 1978). The Chinese Scholar Xiao Shuiyuan conducted in-depth and detailed researches on social support and compiled a social support rating scale. He divided social support into three dimensions, namely subjective support, objective support, and utilization of social support (Xiao, 1994). Previous studies have shown that the family environment could affect the acquisition of individual social support, specifically, a family environment with high intimacy and strong organization was significantly able to improve the level of college students’ acquisition of social support (Wu and Lu, 2006). In the relationship between social support and prosocial behaviors, studies have shown that the level of social support that individuals feel from teachers, peer groups, and family could significantly and positively predict their prosocial behaviors (Qiu and An, 2012). On the basis of the relevant research results, the third hypothesis of this study is proposed, H3: Social support plays a mediating role in the influence of family function on online prosocial behaviors, that is family function can indirectly influence the implementation of online prosocial behaviors through the mediating effect of social support.

According to Eisenberg’s Prosocial Behavior Model Theory, prosocial behaviors could be divided into three stages in line with their psychological change process, that is, the stage of paying attention to the needs of others, the stage of determining the intention to help others, and the stage of linking intention and behaviors (Eisenberg, 2014). To begin with, the first stage to pay attention to others’ need was the initial stage of the implementation of individual prosocial behaviors. At this stage, Eisenberg believed that whether an individual could pay attention to others was affected by two factors, one of which was the relevant individual characteristics, and the second was the individual’s interpretation of a particular situation. The individual factors included individual characteristics formed in the acquired social environment, the parenting style, one’s family function, and so on, which were all important components. And then, the second stage to determine prosocial behaviors intention was divided into two situations, which included the determination of helping intention in emergency situations and the determination of helping intention in non-emergency situations. In emergencies, the critical factors in the decision-making process were emotional factors, such as personal pain, empathy, perspective taking, and guilt; while in non-emergency situations, the individual’s personality traits were the determining factors. Despite under which circumstances, individuals with high empathy ability were more likely to put themselves into the perspective of people who were faced with the events, having stronger emotional involvement and deeper psychological experience, having a relatively more positive attribution and risk–benefit assessment, making it easier to determine the intention of prosocial behaviors. At last, the third stage to establish the connection between intention and behaviors was mainly affected by the individual’s ability to help others and the change between person and the situation (Wang and Pang, 1997). On the basis of Social Learning Theory, which was put forward by behaviorist Bandura, the individual’s performance of certain behavior itself was capable of strengthening one’s own behaviors, and it was often referred as the concept of direct reinforcement. Individuals who received more social support at this stage would have a higher level of self-efficacy in their own abilities, and would feel that they were more competent to put prosocial behaviors into practice. Moreover, positive intimation of self-worth and highly-praised evaluation of others after people implemented prosocial behaviors in the past would also further enhance one’s self-efficacy, and it was much easier to associate the intentions and actions of prosocial behaviors with implement of prosocial behaviors (Guo, 2005). As can be seen in these studies that individuals who had better family function, higher empathy ability, and higher level of social support were more likely to perform online prosocial behaviors. Based on previous empirical research papers and theoretical reasoning, the fourth hypothesis in this study is brought forward, H4: Empathy and social support play a chain mediating role in the influence of family function and online prosocial behaviors. To be specific, individuals with better family function, higher empathy, and higher levels of social support are more likely to develop online prosocial behaviors.

### The role of gender

Relevant studies have shown that gender could moderate the relationship between family function, prosocial behaviors, and online prosocial behaviors (Wang et al., 2020, 2022). The family’s socioeconomic status, parents’ expectations for their children, parents’ own educational experience and life background, as well as clan culture, all of these factors had significant impacts on the parenting style, parents’ attention, and educational investment on their children of different genders (Currie and Moretti, 2007; Ding et al., 2018). Campbell Leaper together with other scholars conducted a four-year follow-up study and found that males were more susceptible to interpersonal interactions and environmental influences than females (Leaper et al., 1989). In the light with Human-situation Interaction Theory, an individual and his situation commonly constituted a whole system (Zeng and Sang, 2005), and individual’s family situation would have an obvious affect on people’s behavioral characteristics when the children’s genders were different, as a result that males and females would have different behavioral response tendencies due to the interaction of family function and gender. Rosa Rosnati and his coworkers recruited 276 Italian families with children aged 11 to 17 as a research sample and found that whether in native or adoptive families, parents’ parenting methods for children of different genders in emotional communication and life interaction events were significantly different (Rosnati et al., 2007), therefore the interaction of family factors and gender can affect people’s behavior patterns and behavioral tendencies, and online prosocial behavior is one of them. Studies have indicated that owning to parents had different parenting goals for males and females and society had different gender requirements for individuals, females might deliberately maintain and strengthen their own prosociality in order to gain the approval of their parents and the acceptance of other people (Wang et al., 2022). In addition, because of the influence of gender stereotypes, family and social acceptance was much higher when males show mischievous actions, while females were always taught to be polite and quiet. Families’ character shaping and expectations of children of different genders in terms of personality were also influential on the frequency of individual prosocial behaviors. Therefore, the fifth hypothesis of this study is proposed, H5: Gender plays a moderating role on the direct path of family function and online prosocial behaviors. In previous studies, some scholars have shown that there were gender differences between males and females in social support (Colarossi, 2001). According to the Gender Schema Theory (Hair et al., 2008) and the expectation of males and females’ gender roles in traditional culture, females were often considered weak and need more support, while males were considered strong, brave and independent, so the level of social support would be different in different genders. Based on what we have talked above, this study puts forward the sixth hypothesis, H6: Gender has a moderating effect on the influence of social support on online prosocial behaviors, that is to say, on the intermediary path from social support to online prosocial behaviors, gender plays a moderating role in both the direct path and the second half path.

Most of the high school students are adolescents, and they are at a very pivotal and relatively special stage in their life (Liu et al., 2020). For one thing, in terms of age, they are in a special transition stage when they have just passed adolescence and then immediately enter into adulthood. For another, in terms of psychology, they are in a special stage of rapid development of self-identity and self-awareness. In the external environment, they are under ardent expectations and enormous pressure from both teachers and parents, and they also face vital tests such as the scores of college entrance examination and academic performance. So is there bullying happening around high school students? Especially in the period of online classes, will cyberbullying occur when online exposure increases? Will they give a helping hand as a bystander with the face of cyberbullying? What factors can promote online prosocial behaviors among high school students? These are what this article is going to study.

## Materials and methods

### Participants

The simple random sampling method was adopted in this study, and questionnaires of the study were gathered in February 2022 in certain normal high schools in Hebei Province and Henan Province in China. The data of this study was distributed and collected online in the form of questionnaires, and the students who were participated in this study used their mobile phones as well as computers to answer the questions independently after class and during vacations. The final size of questionnaires obtained in this study is 1961, and 1861 valid data remained after excluding the invalid ones. The effective ratio of the questionnaire is 94.90%. Among them, 884 are boys (47.5%), and 977 are girls (52.5%); 720 are in the first grade (38.7%), and 645 are in the second grade (34.7%), 496 are in the third grade (26.7%) as well; 167 are the only children in their family (9%), and 1,694 are non-only children (91%); 1710 are from rural areas (91.9%) and 151 are from non-rural areas (8.1%). The average age of the subjects is 16.84 ± 1.08 years; the average Internet age of the subjects is 5.42 ± 2.45 years; during the winter and summer vacations, the time used on Internet is 3.03 ± 0.92 h every day; during the non-winter and summer vacations, the time spent on the Internet is 2.07 ± 1.02 h.

### Family function scale

This study applied the Family Intimacy and Adaptability Scale (FACESII) which was developed by Olson and his colleagues (1982) to measure the subjects’ family function. The scale was modified by Felipeng and his coworkers (Fei et al., 1991) for localization to be suitable for Chinese, which was named Family Intimacy and Adaptability Scale (FACESII-CV). The revised scale has a total number of 30 items, including two dimensions, intimacy and adaptability. The scale uses a five-point Likert scale, ranging from 1 to 5 to indicate “never” to “always.” Participants who gets the higher scores meant that their family has a higher degree of intimacy and adaptability. In this study, the Cronbach’s *α* = 0.814 and 0.846 respectively, and the Cronbach’s *α* = 0.908 of the total scale.

### Interpersonal response indicator scale

This study applied the Interpersonal Response Indicator scale (IRI) which was compiled by Davis (1983), and the scale was localized and revised by Zhang Fengfeng and his coworkers (Zhang et al., 2010) into the Chinese version of the Interpersonal Response Indicator Scale (IRI-C) to measure the subjects’ empathy ability (Zhang et al., 2010). The revised scale has a total number of 22 items, including four dimensions, namely Perspective Taking (PT), Fantasy (FS), Empathy Concern (EC), and Personal Distress (PD). The scale uses a five-point Likert scale, ranging from 0 to 4 to indicate “inappropriate” to “very appropriate.” The higher the score is, the higher the empathy level of the subjects have. In this study, Cronbach’s *α* = 0.783.

### Social support rating scale

In this study, the Social Support Rating Scale (SSRS) developed by Xiao (1994) was used to measure the social support level of the subjects. The scale has a total number of 10 items, including three sub-dimensions, that is, objective support (3 items), subjective support (4 items), and utilization of social support (3 items). The final scores less than 20 represent “low level social support,” scores between 20 and 30 represent “medium level social support,” and scores greater than 30 represent “high level social support” (Liu et al., 2020). In this study, the mean score of the subjects’ social support level was 39.01, which is a high level of social support. In this study, Cronbach’s *α* = 0.727.

### Questionnaire on bystander behavior in cyberbullying

In this study, the Bystander Behavior Questionnaire in Cyberbullying developed by Teng (2015) was used to measure the subjects’ reactions to cyberbullying. The scale consists of 20 items and includes three sub-dimensions, namely, behaviors that promote bullying (7 items), behaviors that protect the bullied (9 items), and outsider behaviors (4 items). The scale is scored on a seven-point scale, ranging from 1 to 7 to indicate “completely disagree” to “totally agree.” The scale is scored on three sub-dimensions respectively, and the higher the score is, the higher the tendency of the subjects to approach this behavior is. In this study, Cronbach’s *α* = 0.927, 0.958, and 0.904, respectively.

### Statistical analysis and common method bias test

This study used SPSS 21.0 to perform descriptive statistics, *t*-test, and correlation analysis on the collected data, and used the PROCESS macro program of Hayes (2013) to test and analyze mediating and moderating effects. Since data were collected in a self-reported manner, results may be subject to common method biases (Zhou and Long, 2004). In this study, in order to control the confusion of the research results caused by the common method bias, in the aspect of program control, it was firstly stated in the instruction setting of the test questionnaire that this questionnaire will be filled in anonymously, and the answers to the questionnaire will be strictly confidential and the answers will only be for academic research (Hu et al., 2019). Secondly, the subjects who participated in the study came from different provinces and cities such as Hebei Province and Henan Province, and were selected from different school levels and school types. As for the term of statistical control, Harman’s Univariate Method was used to test for common method bias in this study. The results showed that the eigenvalues of 11 factors were greater than 1 in total, and the variance explained by the largest factor was 19.94%, which was less than 40%, indicating that there was no serious common method bias effect in this study (Xiong et al., 2012).

## Results

### Gender and grade differences

Gender differences in family function, empathy, social support, and cyberbullying bystander behaviors were investigated by independent-samples t-test statistical methods in this study. The results showed that there are significant differences in the other variables except for the variable of social support, which had no significant gender difference. There was a significant gender difference in family function (*t* = 2.76, *p* < 0.01). Compared with females, males had a higher level of family function. There was a significant gender difference in empathy (*t* = −5.76, *p* < 0.001). Compared with males, females had a higher level of empathy. There was a significant gender difference in the behavior of promoting bullying (*t* = 2.91, *p* < 0.01), and males had more bullying-promoting behaviors compared with females. There was a significant gender difference in the behavior of protecting the bullied (*t* = −2.57, *p* < 0.05). Specifically, females had more behaviors to protect the bullied compared with males. There was a significant gender difference in bystander behavior (*t* = 2.98, *p* < 0.01). Compared with females, males have more bystander behaviors.

The differences caused by grade in family function, empathy, social support, and cyberbullying bystander behaviors were investigated by ANOVA test statistical methods in this study. The results showed that only the variable of empathy ability had a significant difference in grades (*F* = 4.648, *p* < 0.05). The LSD post-hoc test showed that the empathy ability of Grade 1 had the highest score, and the empathy ability of Grade 3 was the lowest. And except for empathy, no significant differences in grades were found in other dimensions in this study (See Table 1).

**Table 1:** Gender and grade differences.

	Male (*N* = 884)		Female (*N* = 997)		*t*	Grade 1 (*N* = 720)		Grade 2 (*N* = 645)		Grade 3 (*N* = 496)		*F*
	*M*	*SD*	*M*	*SD*		*M*	*SD*	*M*	*SD*	*M*	*SD*	
FF	144.43	19.38	142.04	17.87	2.76^**^	142.51	18.35	143.08	18.41	144.27	19.31	1.318
E	48.89	10.94	51.78	10.70	−5.76^***^	51.04	11.37	50.66	10.5	49.16	10.66	4.648^*^
SS	39.02	6.45	39.01	5.96	0.04	39.09	6.12	38.9	6.24	39.06	6.26	0.178
BPromoteB	13.85	9.89	12.57	8.95	2.91^**^	12.88	9.39	13.06	9.17	13.76	9.8	1.362
BProtectB	42.73	15.83	44.47	13.05	−2.57^*^	44.43	14.60	43.41	14.39	42.79	14.32	2.021
BStanderB	12.61	6.57	11.74	5.95	2.98^**^	11.84	6.30	12.12	6.05	12.64	6.46	2.406

FF, Family function; E, Empathy; SS, Social support; BPromoteB, Bullying-promoting behavior; BProtectB, Bullying-protecting behavior; BStanderB, Bystander behavior. ^***^p < 0.001; ^**^p < 0.01; ^*^p < 0.05.

### Correlations among all variables

The study applied Pearson Product–moment Correlation to test the correlations among all variables. It was found that except for few dimensions, all dimensions basically showed significant pairwise correlations. Family function was significantly positively correlated with empathy, social support, bullying-promoting behavior, and bullying-protecting behavior, and significantly negatively correlated with bystander behavior. Empathy was significantly positively correlated with social support and bullying-protecting behavior, and negatively correlated with bullying-promoting and bystander behavior. Social support was significantly positively correlated with bullying-protecting behavior, and negatively correlated with bystander behavior. Bullying-promoting behavior was significantly positively correlated with bullying-protecting behavior and bystander behavior. Bullying-protecting behavior was significantly negatively correlated with bystander behavior (See Table 2).

**Table 2:** Descriptive statistics and correlation coefficient matrix for each variable (*N* = 1861).

	*M*	*SD*	1	2	3	4	5	6
1. FF	143.18	18.64	1					
2. E	50.41	10.91	0.24^**^(0.00)	1				
3. SS	39.01	6.20	0.44^**^(0.00)	0.16^**^(0.00)	1			
4. BPromoteB	13.18	9.43	0.05^*^(0.02)	−0.07^**^(0.01)	0.04	1		
5. BProtectB	43.64	14.46	0.22^**^(0.00)	0.26^**^(0.00)	0.33^**^(0.00)	0.06^*^(0.02)	1	
6. BStanderB	12.15	6.26	−0.14^**^(0.00)	−0.21^**^(0.00)	−0.24^**^(0.00)	0.18^**^(0.00)	−0.32^**^(0.00)	1

FF, Family function; E, Empathy; SS, Social support; BPromoteB, Bullying-promoting behavior; BProtectB, Bullying-protecting behavior; BStanderB, Bystander behavior. ^***^p < 0.001; ^**^p < 0.01; ^*^p < 0.05.

### Chain mediating effects of empathy and social support between family function and bullying-protecting behavior

According to the results of the correlation analysis in this study and the statistical preconditions of the mediation effect, further mediation effect analysis of empathy and social support can be carried out (Wen and Ye, 2014). In order to study the role of empathy and social support in family function and bullying-protecting behavior in the face of cyberbullying, the study used the bias-corrected percentile Bootstrap method in the SPSS macro program Process compiled by Hayes (2013) to analyze the mediating effect (Fang et al., 2015), and Model 6, which specialized in analyzing chain mediation effects, was used for testing. This study used a Bootstrap sample size of 5,000 times to test the chain mediation effect of empathy and social support with a 95% confidence interval. Among the demographic variables in this study, the proportion of students’ origin and whether they were only children was quite different, and the grade factor had little effect on the variables to be studied in the previous ANOVA analysis, so these factors were not controlled in the next tests. However, in the independent sample t-test on sex, it was found that most variables in this study had significant differences on it. In order to avoid the error caused by this factor on the research results, gender was used as a covariate to control for the chain mediation effect test.

The results of regression analysis showed that family function significantly and positively predicted the bullying-protecting behaviors (*β* = 0.172, *p* < 0.001) and empathy (*β* = 0.146, *p* < 0.001). After incorporating social support into the regression equation of family function and empathy, the results suggested that family function significantly and positively predicted social support (*β* = 0.141, *p* < 0.001), and empathy also significantly as well as positively predicted social support (*β* = 0.031, *p* < 0.05). And then, after incorporating the bullying-protecting behaviors into the regression equation of family function, empathy, and social support, it was indicated that family function significantly and positively predicted the bullying-protecting behaviors (*β* = 0.044, *p* < 0.05), and empathy also significantly and positively predicted the bullying-protecting behaviors (*β* = 0.256, *p* < 0.001), and social support also significantly and positively predicted the bullying-protecting behaviors (*β* = 0.647, *p* < 0.001) as well (See Table 3).

**Table 3:** Model for regression analysis between variables (*N* = 1861).

Outcome variable	Predictor variable	*R*	*R^2^*	*F*	*β*	*t*
BProtectB		0.221	0.049	96.687^***^		
	FF				0.172	9.777^***^
E		0.282	0.080	80.454^***^		
	FF				0.146	11.201^***^
SS		0.440	0.193	148.444^***^		
	FF				0.141	19.646^***^
	E				0.031	2.517^*^
BProtectB		0.396	0.157	86.073^***^		
	FF				0.044	2.320^*^
	E				0.256	8.684^***^
	SS				0.647	11.684^***^

FF, Family function; E, Empathy; SS, Social support; BProtectB, Bullying-protecting behavior. ^***^p < 0.001; ^**^p < 0.01; ^*^p < 0.05.

**Figure 1:**
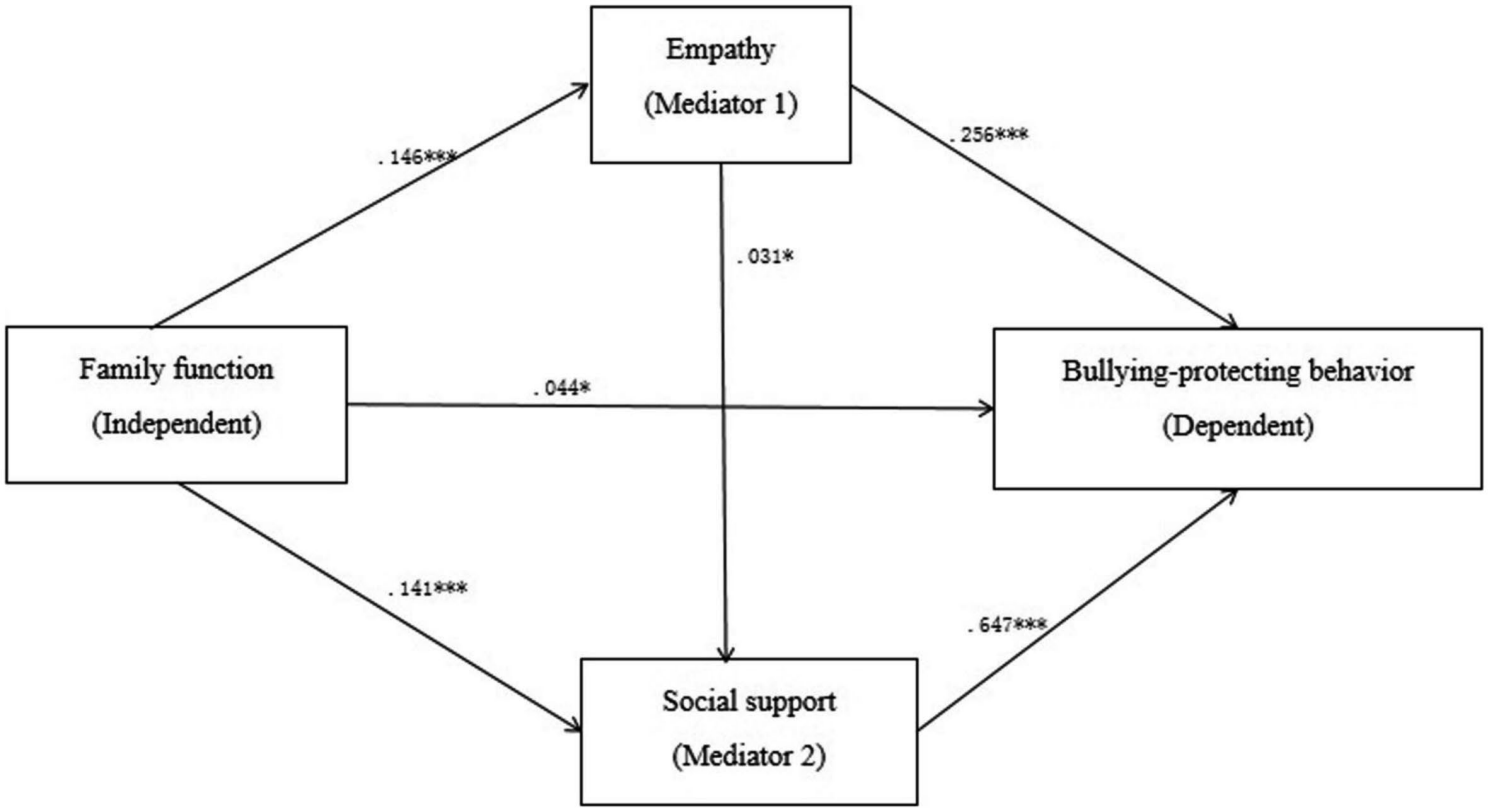
The mediating role of empathy and social support between family functioning and bullying-protecting behavior.

The results of the mediation effect analysis showed that empathy and social support played a significant mediating role between family function and the bullying-protecting behavior. The total mediation effect value was 0.132 (Boot 95% CI = [0.109, 0.156]), accounting for 75.43% of the total effect of family function on the bullying-protecting behavior. Specifically, the mediating effect of family function and the bullying-protecting behavior is composed of indirect effects generated by three paths, path 1:family function → empathy →the bullying-protecting behavior (effect value 0.038, Boot 95% CI = [0.027, 0.050]); Path 2: family function → empathy → social support → the bullying-protecting behavior (effect size 0.003, Boot 95% CI = [0.001, 0.006]); Path 3: family functioning→social support→the bullying-protecting behavior (effect size 0.091, Boot 95% CI = [0.072, 0.112]). The ratios of the indirect effects of the three pathways to the total effects were 21.71%,1.71%, and 52.01%, respectively. Moreover, the 95% confidence intervals of the three indirect effects did not contain 0 values, indicating that the three indirect effects reached a significant level. Use the options in Model 6 of Process to compare indirect effects, compare three different indirect path effects in pairs, and explore whether there are significant differences among them. The results showed that, comparison 1 showed that the Bootstrap 95% confidence interval of the difference between indirect effect 1 and indirect effect 2 did not contain 0 value, indicating that there was a significant difference between indirect effect 1 and indirect effect 2; Comparison 2 showed that the Bootstrap 95% confidence interval for the difference between indirect effect 1 and indirect effect 3 did not contain 0 value, indicating that there was a significant difference between indirect effect 1 and indirect effect 3; Comparison 3 showed that the Bootstrap 95% confidence interval for the difference between indirect effect 2 and indirect effect 3 did not contain 0 value, indicating that there was a significant difference between indirect effect 2 and indirect effect 3 (See Table 4 and Figure 1).

**Table 4:** Mediating effect size analysis (*N* = 1861).

	Indirect effect size	Boot SE	Boot LLCI	Boot ULCI	Relative mediation effect
Total indirect effect	0.132	0.012	0.109	0.156	75.43%
Indirect effect 1	0.038	0.006	0.027	0.050	21.71%
Indirect effect 2	0.003	0.001	0.001	0.006	1.71%
Indirect effect 3	0.091	0.010	0.072	0.112	52.01%
comparison 1	0.035	0.006	0.024	0.048	
Comparison 2	−0.054	0.012	−0.077	−0.031	
Comparison 3	−0.088	0.010	−0.109	−0.070	

Boot SE, Standard Error of Indirect Effects; Boot LLCI, The lower bound of the 95% confidence interval; Boot ULCI, The upper limit of the 95% confidence interval (Percentile Bootstrap Method with Bias Correction). Indirect effect 1: family function—empathy—online prosocial behaviors; Indirect effect 2: family function—social support—online prosocial behaviors; Indirect effect 3: family function—empathy—social support—online prosocial behaviors. Comparison 1, Difference Between Indirect Effect 1 and Indirect Effect 2; Comparison 2, Difference Between Indirect Effect 1 and Indirect Effect 3; Comparison 3, Difference Between Indirect Effect 2 and Indirect Effect 3. All values are rounded to 3 decimal places.

### Analysis of the moderating effect of gender among family function, empathy, and the bullying-protecting behavior

Equation 1 According to the above test, it can be shown that empathy and social support have a mediating effect between family function and the bullying-protecting behavior. However, the effect value of indirect effect 2 (family function → empathy → social support → bullying-protecting behavior) is relatively low (1.71%). Therefore, we will only explore the moderating effect of gender on the two mediating effect pathways of indirect pathway 1 (family function → empathy → bullying-protecting behavior) and indirect pathway 3 (family function → social support → bullying-protecting behavior). According to the moderated mediation model test method suggested by Wen and Ye (2014), this study first standardized all variable data, and coded the gender variable as a dummy variable (1 for male and 0 for female). Next, the Process program was used to test the moderated mediation model with family function as the independent variable, empathy as the mediator variable, gender as the moderator variable, and the bullying-protecting behavior as the dependent variable. The results showed that family function had a significant positive predictive effect on empathy (*β* = 0.250, *t* = 11.156, Boot 95% CI = [0.205, 0.293], *p* < 0.001); Gender had a significant negative predictive effect on empathy (*β* = −0.287, *t* = −6.420, 95% CI = [−0.374, −0.200], *p* < 0.001); The interaction term of family function and gender had no significant effect on the prediction of empathy (*β* = 0.076, *t* = 1.700, 95% CI = [−0.012, 0.163]) (See Table 5 Equation 1).

**Table 5:** Analysis of the moderating effect of sex on the mediating effect of empathy in bullying-protecting behavior.

Predictor variable	Equation 1 E(M)			Equation 2 BProtectB(Y)		
	*B*	*SE*	95%CI	*B*	*SE*	95%CI
Grade	−0.043	0.028	[−0.097, 0.111]	−0.053	0.027	[−0.107, 0.001]
Age	−0.046	0.028	[−0.100, 0.008]	0.027	0.027	[−0.027, 0.081]
FF	0.250^***^	0.022	[0.205, 0.293]	0.177^***^	0.023	[0.132, 0.222]
Gender	−0.287^***^	0.045	[−0.374, −0.200]	−0.086	0.045	[−0.174, 0.002]
FF × Gender	0.076	0.045	[−0.012, 0.163]	−0.103^*^	0.046	[−0.193, −0.013]
E				0.208^***^	0.023	[0.163, 0.254]
E × Gender				0.075	0.046	[−0.015, 0.165]
*R* ^2^	0.087			0.099		
*F*	35.488^***^			29.171^***^		

FF, Family function; E, Empathy; SS, Social support; BProtectB, Bullying-protecting behavior. ^***^p < 0.001; ^**^p < 0.01; ^*^p < 0.05.

Family function had a significant positive predictive effect on the bullying-protecting behavior (*β* = 0.177, *t* = 7.731, Boot 95% CI = [0.132, 0.222], *p* < 0.001); The interaction item of family function and gender had a significant predictive effect on the bullying-protecting behavior (*β* = −0.103, *t* = −2.250, Boot 95% CI = [−0.193, −0.013], *p* < 0.05); Empathy had a significant positive predictive effect on the bullying-protecting behavior (*β* = 0.208, *t* = 9.026, Boot 95% CI = [0.163, 0.254], *p* < 0.001); The interaction term of empathy and gender had no significant effect on the bullying-protecting behavior (*β* = 0.075, *t* = −1.629, Boot 95% CI = [−0.015, 0.165]). The results of this model verified that empathy mediated between family function and the bullying-protecting behavior, and that the direct pathway of this model is moderated by gender (See Table 5 Equation 2).

**Figure 2:**
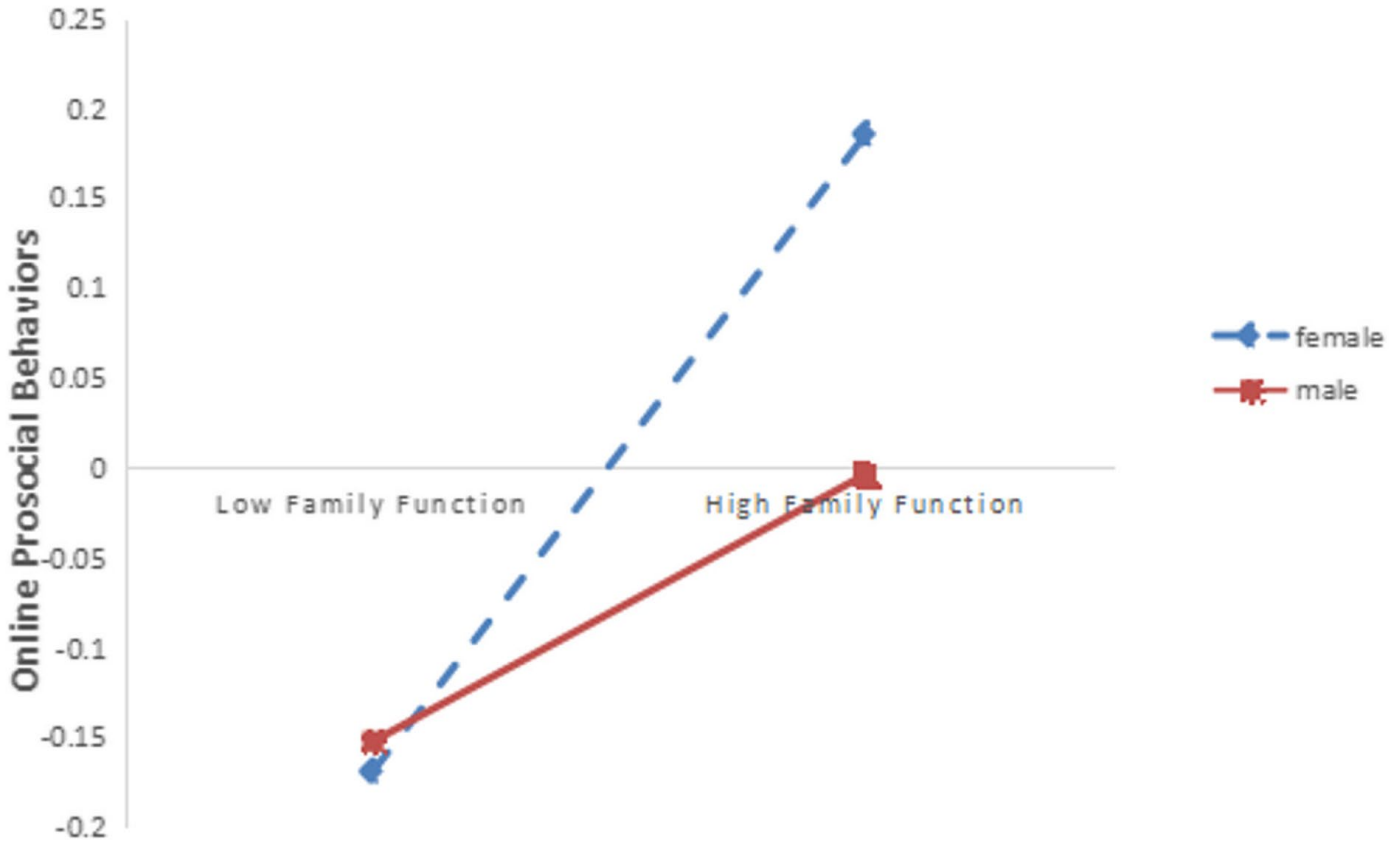
The interaction of family function and gender on online prosocial behaviors (the mediating variable is empathy).

The method of simple slope analysis was used to further analyze the moderating effect of gender when empathy was the mediating variable of family function and high school students’ online prosocial behavior. The results showed that in the male group, family function had a significant effect on predicting online prosocial behavior (simple slope = 0.123, *t* = 7.000, *p* < 0.001); in the female group, family function also had a significant predictive effect on online prosocial behavior (simple slope = 0.226, *t* = 3.820, *p* < 0.001). However, the effect sizes of the two groups were different, indicating that gender can play a moderating role between family function and online prosocial behavior (See Figure 2).

### Analysis of the moderating effect of gender among family function, social support, and the bullying-protecting behavior

Equation 1 the Process program macro was used to test the moderated mediation model with family function as the independent variable, social support as the mediator variable, gender as the moderator variable, and the bullying-protecting behavior as the dependent variable, as well as grade and age as covariates. The results showed that family function had a significant positive predictive effect on social support (*β* = 0.436, *t* = 20.827, Boot 95% CI = [0.396, 0.479], *p* < 0.001); the interaction term of family function and gender had no significant predictive effect on social support (*β* = 0.018, *t* = 0.434, Boot 95% CI = [−0.046, 0.100]) (See Table 6 Equation 1).

**Table 6:** Analysis of the moderating effect of sex on the mediating effect of social support in bullying-protecting behavior.

Predictor variable	Equation 1 SS(M)			Equation 2 BProtectB(Y)		
	*B*	*SE*	95%CI	*B*	*SE*	95%CI
Grade	−0.021	0.026	[−0.071, 0.031]	−0.056	0.027	[−0.108, 0.003]
Age	0.003	0.026	[−0.048, 0.054]	0.016	0.027	[−0.037, 0.070]
FF	0.436^***^	0.021	[0.396, 0.479]	0.106^***^	0.024	[0.059, 0.154]
Gender	−0.054	0.042	[−0.136, 0.030]	−0.132^**^	0.044	[−0.217, −0.046]
FF × Gender	0.018	0.042	[−0.064, 0.100]	−0.149^**^	0.048	[−0.243, −0.054]
SS				0.283^***^	0.024	[0.059, 0.154]
SS × Gender				0.172^***^	0.048	[0.077, 0.266]
*R* ^2^	0.191			0.132		
*F*	87.654^***^			40.181^***^		

FF, Family function; E, Empathy; SS, Social support; BProtectB, Bullying-protecting behavior. ^***^p < 0.001; ^**^p < 0.01; ^*^p < 0.05.

Family function had a significant positive predictive effect on the bullying-protecting behavior (*β* = 0.106, *t* = 4.382, Boot 95% CI = [0.059, 0.154], *p* < 0.001); Gender had a significant negative predictive effect on the bullying-protecting behavior (*β* = −0.132, *t* = −0.319, Boot 95% CI = [−0.217, −0.046], *p* < 0.005); The interaction term of family function and gender had a significant predictive effect on the bullying-protecting behavior (*β* = −0.149, *t* = 3.081, Boot 95% CI = [−0.243, −0.054], *p* < 0.005); Social support had a significant positive predictive effect on the bullying-protecting behavior (*β* = 0.283, *t* = 4.383, Boot 95% CI = [0.059, 0.154], *p* < 0.001); The interaction term of social support and gender had a significant predictive effect on the bullying-protecting behavior (*β* = 0.172, *t* = 3.566, Boot 95% CI = [0.077, 0.266], *p* < 0.005). The results of this model verified that social support mediated the relationship between family function and the bullying-protecting behavior, and that the direct pathway and the second half pathway of this model are moderated by gender (See Table 6 Equation 2).

**Figure 3:**
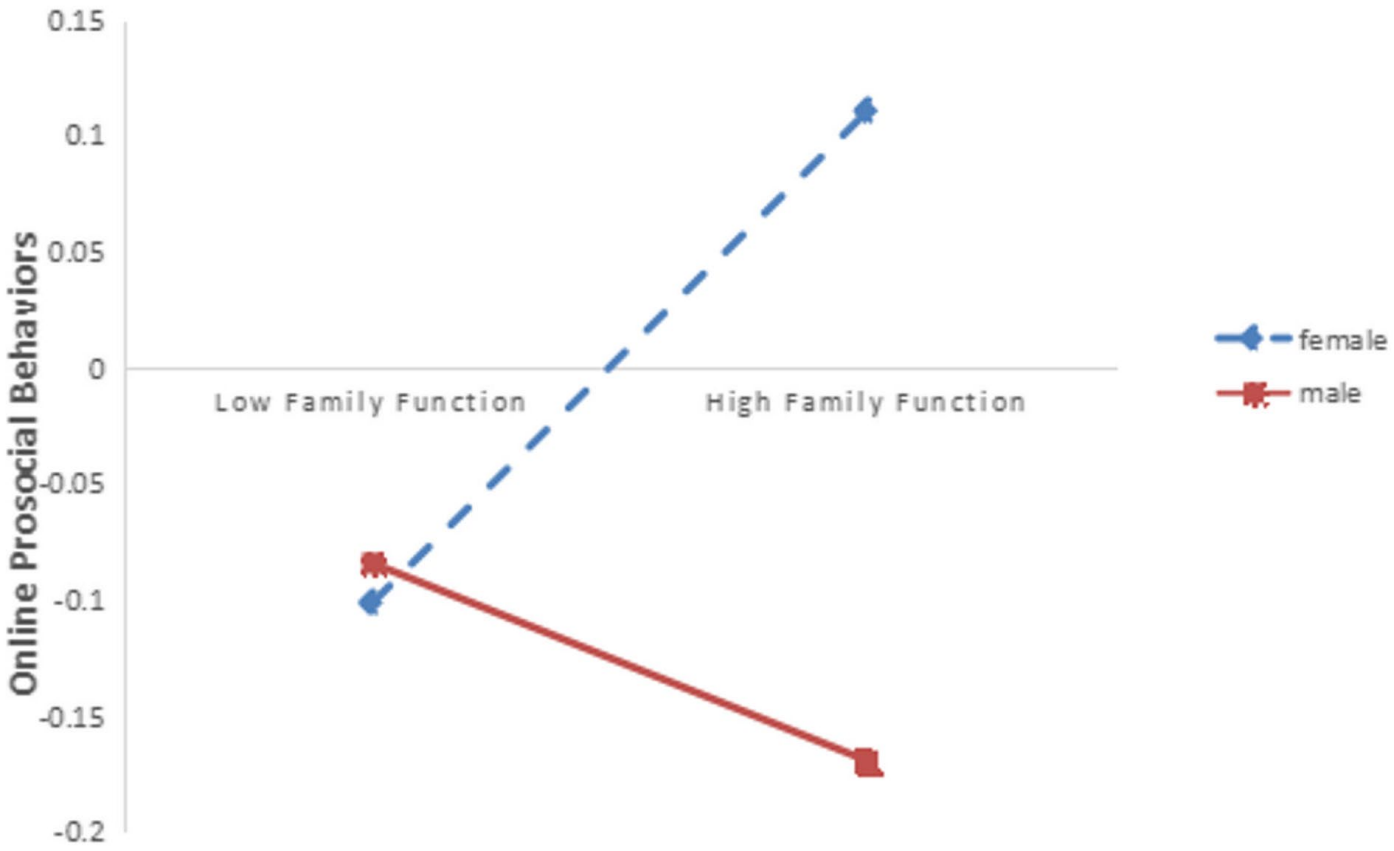
The interaction of family function and gender on online prosocial behaviors (the mediating variable is social support).

The method of simple slope analysis was used to further analyze the moderating effect of gender when social support was the mediating variable of family function and high school students’ online prosocial behavior. A simple slope plot was used to determine the differences in the influence of family function on online prosocial behaviors for different genders. The results showed that in the male group, family function had no significant effect on predicting online prosocial behavior (simple slope = 0.280, *t* = 0.829, *p* > 0.05); In the female group, family function had a significant effect on the prediction of online prosocial behavior (simple slope = 0.177, *t* = 5.124, *p* < 0.001), indicating that gender can play a moderating role between family function and online prosocial behavior (See Figure 3).

**Figure 4:**
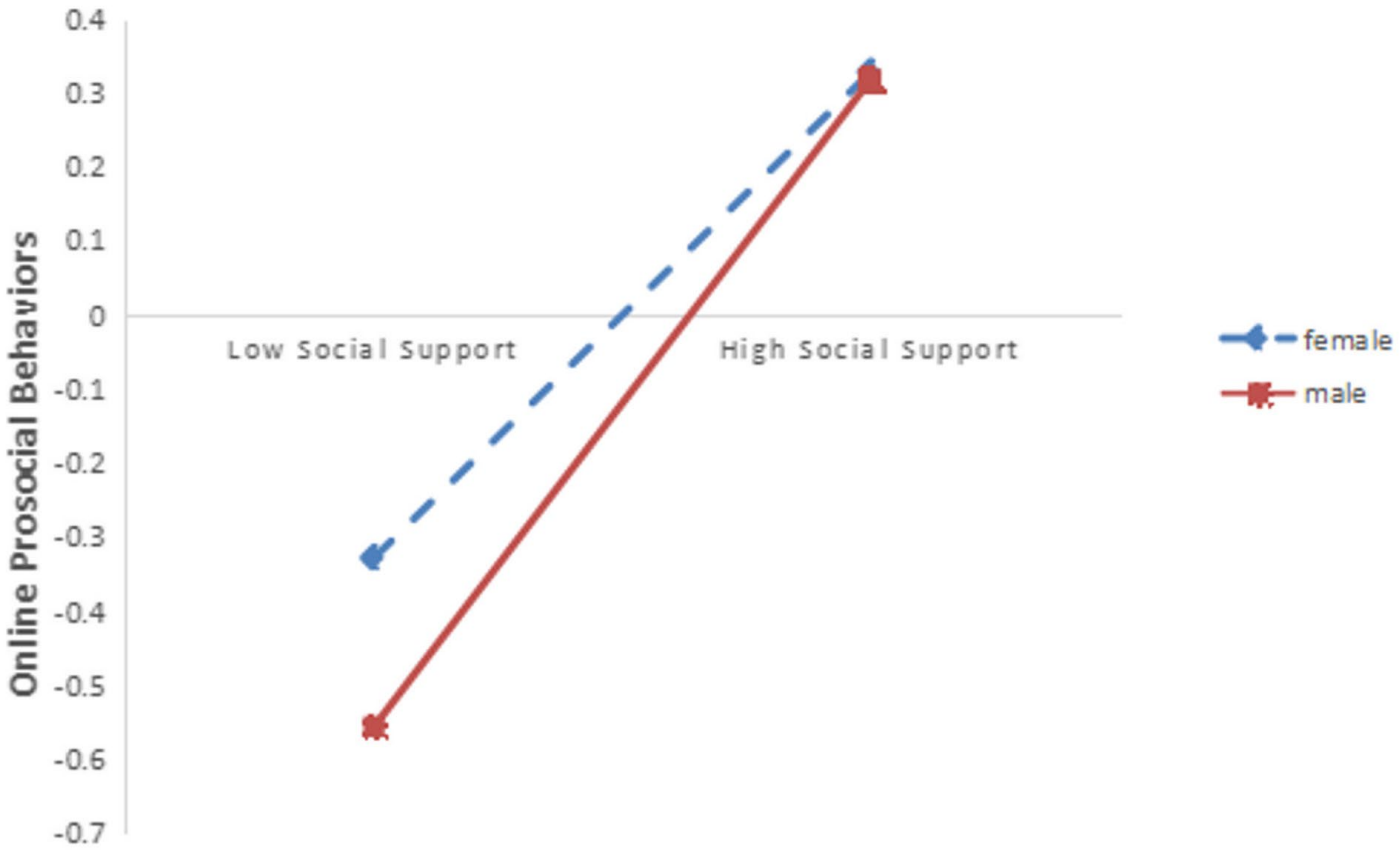
The interaction of social support and gender on online prosocial behaviors (the mediating variable is social support).

The method of simple slope analysis was used to further analyze the moderating effect of gender between social support and online prosocial behavior of high school students. A simple slope plot was used to determine the differences in the influence of social support on online prosocial behavior for different genders. The results showed that in the male group, social support had a significant effect on predicting online prosocial behavior (simple slope = 0.385, *t* = 12.686, *p* < 0.001); In the female group, social support also had a significant predictive effect on online prosocial behavior (simple slope = 0.276, *t* = 8.837, *p* < 0.001), indicating that gender can play a moderating role between social support and online prosocial behavior (See Figure 4).

## Discussion

This study mainly discussed the chain mediating pathway of family function and online prosocial behaviors of high school students as bystanders of cyberbullying incidents. The results indicated that family function influenced online prosocial behaviors through the indirect pathways of empathy, social support, and the chain mediating pathway of empathy and social support, as well as that gender moderated the two indirect pathways.

### The predictive effect of family function on online prosocial behavior

This study investigated the relationship between family function and high school students’ online prosocial behaviors with the face of cyberbullying, and found that family function could directly and positively predict high school students’ online prosocial behaviors, which confirmed the first hypothesis of this study. Consistent with previous research findings, positive parenting, high levels of parent–child relationships, and good family function could promote the implementation of individual’s prosocial behaviors (Pastorelli et al., 2016). According to Bronfenbrenner’s Ecological Systems Theory, the innermost micro-system of the environmental level was the direct environment of high school students’ interactions and activities. So the parenting styles in the family environment, parents’ expectations, family intimacy, and adaptability all played essential roles in directly affecting people’s social behaviors (Ungar et al., 2013; Zhou et al., 2020). In light with The Family Circumplex Model proposed by Olson, family function was divided into two indicators, family intimacy, and family adaptability (Beavers and Voeller, 1983). Studies have shown that family intimacy made a difference to promoting people’s prosocial behaviors (Li et al., 2020), and family adaptability also positively predicted adolescents’ school participation, actively making friends and helping conducts (Annunziata et al., 2006). The Theory of Marriage and Family Function which was put forward by Chinese scholar Fei Xiaotong believed that people’s family was the first place for individual’s socialization. The family shaped children’s social roles, taught individual’s social norms, formulated their life goals, and cultivated individual socialization to become a social person and integrate into the society (Pan, 2010). High school students with good family function received more company time (Dou et al., 2022), financial and psychological support, reduced their anxiety, interacted more favorably with others, and were more likely to engage in prosocial behaviors (Chen et al., 2021). Therefore, online prosocial behaviors, which acted as an unmissable component of individual socialization behaviors, the quality of one’s family function had a very significant impact on it.

### The mediating role of empathy in the influence of family function on online prosocial behavior

This study found that empathy played a mediating role between family function and high school students’ online prosocial behaviors. Specifically, family function positively predicted an individual’s empathy ability, thereby further promote online prosocial behaviors, which was consistent with previous research results (Fan et al., 2020), and verified the second hypothesis of this study. This indirect effect accounted for 21.71% of the total effect. According to Davis’s Multi-dimensional Theoretical Construction based on empathy, empathy included two orientations, cognitive empathy and emotional empathy (Davis, 1983). Referring to the Theoretical Model of Empathy’s Life-long Development, emotional empathy was greatly affected by innate factors, and cognitive empathy was significantly affected by the acquired environment (Decety and Svetlova, 2012). Therefore, family environment which acted as an important acquired factor for individuals was of great importance to one’s empathy. In light with the Empathy-altruism Hypothesis put forward by Batson, the greater the emotional intensity of a person’s empathy was, the higher the motivation for altruistic behaviors was, and the easier it was to carry out altruistic behaviors (Batson et al., 1991; Zhang et al., 2020).

### The mediating role of social support in the influence of family function on online prosocial behavior

This study found that empathy and social support played a chain mediating role between family function and high school students’ online prosocial behaviors. Specifically, family function first worked on empathy, then empathy affected social support, and finally social support acted on online prosocial behaviors, forming the path of “family function—empathy—social support—online prosocial behaviors.” Because the mediation path of “family function—empathy—online prosocial behaviors” had been discussed before, the next part will be discussed from the mediation effect chains “family function—social support—online prosocial behaviors” and “family function—empathy—social support—online prosocial behaviors” separately.

This study found that family function would have an indirect effect on high school students’ online prosocial behaviors through social support, which confirmed the third hypothesis of this study. This indirect effect accounted for 52.01% of the total effect. Moreover, a strong connection between family function and social support had been supported in many previous studies (Bokhorst et al., 2010). According to the Reciprocity Theory of Altruistic Behavior proposed by Trivers, the altruistic behaviors between individuals were mutual, and the social support that an individual received would have an important impact on his altruistic behaviors. Generally speaking, the more social support an individual felt, the more altruistic behaviors people would perform (Helsen et al., 2000). Based on Bandura’s Social Learning Theory (Yildirim et al., 2020), an important factor that promoted or inhibited people’s prosocial behaviors was the observation of the practices of people around you, that is, the concept of indirect reinforcement. Overall, individuals with good family functions had a higher degree of harmony in family relationships, and the mutual aid behaviors shown by family members would provide more mental power, psychological safety, and support to each other, including material support, spiritual support, emotional support, and so on. In addition, individuals living in an environment with good family function, family intimacy and adaptability would also perceive more social support from the family, thus showing more prosocial behaviors. Moreover, according to Deutsch and Lamberti, when the helping behaviors implemented by individuals were reinforced by gratitude, praise, and other positive feedback, individuals would be more inclined to show higher frequency of prosocial behaviors (Deutsch and Lamberti, 1986). A well-functioning family environment was an environment full of encouragement, affirmation, recognition, positive atmosphere, and appreciation, which would make individuals feel more social support for their prosocial behaviors, thereby further strengthening their prosocial behavioral motivation and implementation of prosocial behaviors.

### The chain mediating role of empathy and social support in the influence of family function on online prosocial behavior

This study also found that family function could also have an indirect effect on high school students’ online prosocial behaviors through the chain mediating effect of empathy and social support, which verified hypothesis 4 of this study. This indirect effect accounted for 1.71% of the total effect. Chinese scholar Xianliang Zheng, in his book *The Theory and empirical Research on Internet Altruistic Behavior*, believed that the influencing factors of Internet altruistic behaviors were mainly involved in three aspects, namely factors of helpers, helping seekers, and network environment (Zheng, 2013). In the helper factor, the helper’s family environment and family function had a significant impact on individual’s acquired personality quality, and the individual’s empathy ability was an important part of these qualities. As for the helping seeker factor, the homogeneity of the helping seekers and the potential helpers was closely related to whether to perform the helping behaviors or not. Individuals with strong empathy were capable of perceiving and experiencing the unfavorable situation of the online bullied people, psychologically enhancing the homogeneity of potential helpers and helping seekers. In the network environment, due to the various characteristics of the Internet, it was easier for individuals to carry on network altruistic behaviors (Guo and Wang, 2010). For example, the anonymity of the Internet gave the helping seekers greater courage to self-disclose, and the potential helpers could better understand the situation of the seekers, which increased the possibility of giving social support to them. The timeliness of Internet made the implementation of relational online altruistic behaviors more rapid, and the conducts of online helpers could be reflected in the events of the helper in a timely manner. The interactive nature of the Internet made it more efficient for individuals to implement online prosocial behaviors as bystanders of cyberbullying accidents (Cheng, 2002). According to Erickson’s Theory of Social Personality Development Stages, the main contradiction for high school students was the contradiction between individual role identity and role confusion. The core problem faced by high school students at this stage was the determination of self-awareness and self-role formation. Self-identity could help high school students coordinate the relationship between various people and surrounding matters, and make the transition to adulthood smoothly (Erikson, 1994). High school students with good family functions have developed higher empathy abilities, and their ability to empathize made individuals more friendly to those around them, meanwhile it was easier to obtain higher quality of friendship and social support. In Batson’s view to analyze people’s characteristics and motivation of prosocial behaviors, individuals with high social support actively implemented online prosocial behaviors so as to relieve others’ troubles and help others solve their problems. At the same time, they would also further gain the positive evaluations of themselves and others, even get rewards, social approval, and reduce aversive arousal (Batson et al., 1991). As a result, helpers could obtain higher self-efficacy and a higher sense of self-identity, solve the psychological development contradictions faced by high school students during adolescence, and achieve the purpose of improving the level of individual physical and mental health development.

### The moderating role of gender in family function-empathy-online prosocial behavior

The study found that when the variable of gender was introduced into the study of family function and senior high school students’ online prosocial behaviors, then the results showed that gender played a moderating role in both the pathways of family function-empathy-online prosocial behaviors and family function-social support-online prosocial behaviors, which was consistent with previous research results (Earnshaw et al., 2011; Brown et al., 2014), and verified the fifth hypothesis of this study. Based on the results in pathway of family function-empathy-online prosocial behaviors, it could be found that there were interactions between family function and gender. Compared with males, females with a high family function level were more likely to perform online prosocial behaviors. In conformity with the Gendered Family Process Model, there were different ways for parents to raise males and females, as a result, the differences between education methods and family functions would shape the differences in their characteristics, social communication styles, and behavior tendencies of males and females (Endendijk et al., 2018). Generally speaking, females would be educated by parents to be kind, benevolent, and helpful, so it was easier for them to extend a helping hand in the face of cyberbullying. By contrast, the orientation of males’ family education was supposed to be reserved, stable, restrained, and unassuming. Therefore, when males found that others were in the state of being bullied online, they were more likely to turn a blind eye and remain silent. Therefore, family functions showed interactions on different genders, and gender played a moderating role in the direct pathway from family functions to online prosocial behaviors.

### The moderating role of gender in family function-social support-online prosocial behavior

On the intermediary path of family function-social support-online prosocial behaviors, it could find that gender not only played a moderating role in the direct path, but also played a moderating role in the second half of the path. This result verified the sixth hypothesis of this study. On one hand, in the direct path of mediating family function and online prosocial behavior with social support, females with high family function were more likely to implement online prosocial behavior than males. According to the Relational Theory put forward by Portman, in comparison with adolescent males, adolescent females matured earlier than males in both physical and psychological aspects (Portman et al., 2010), so the surrounding environmental factors and the family influence they have received would also have a greater impact on females. Family function which acted as an important part of environmental factors, would also have a greater impact on females, making family function interact with sex, so that the females who had higher level family function showed a higher level of online prosocial behaviors. On the other hand, compared with females, males who received high-level social support were more likely to show online prosocial behaviors. Compared with females who were introverted and gentle, males were more extroverted and strong. In addition, no matter the knights saved the princess in western culture or the heroes saved the beauty in eastern culture, under the expectation of social roles, males are endowed with higher role expectations in helping others than females. Therefore, when they received a high level of social support, they were more determined to act as protectors and messengers of justice, and were more likely to carry out online prosocial behavior than females.

In summary, all the six research hypotheses in this study have been verified. Family function of high school students had a direct predictive effect on online prosocial behaviors. Empathy and social support not only played a mediating role, respectively, in the influence of family function on online prosocial behaviors, but also played a chain mediating role between them. Therefore, two mediation models and one chain mediation model were obtained. In the mediating model of family function-empathy-online prosocial behaviors, gender played a moderating role in the direct pathway. In the mediating model of family function-social support-online prosocial behaviors, gender played a moderating role in the direct path and the latter half path, respectively.

## Limitation

This study also had certain limitations. To begin with, from the perspective of research methods, this research was based on the previous theoretical constructions and research models, which adopted a cross-sectional research method through questionnaires and scales to conduct the research. Yet this research design failed to explore the causal relationship between independent variable and dependent variable Future research can design experiments or use cross-lagged studies to further explore the causal relationship between them. In addition, the online prosocial behaviors of high school students measured in this study were only one of the responses of high school students when they faced cyberbullying situations, so little attention was paid to whether they would promote cyberbullying or turn a blind eye. Future research can also focus on other varieties of responses of high school students in the face of cyberbullying and its impact mechanism.

## Conclusion

This study found that family function positively predicted high school students’ online prosocial behaviors, in which empathy and social support played a chain mediating role between them, and gender moderated this mediation model. This paper further revealed the mechanism of family function on high school students’ online altruistic behaviors, enriching the research on high school students’ online prosocial behaviors. And it can provide empirical and theoretical basis for cultivating high school students’ online prosocial behaviors in family, school, and society.

## Data availability statement

The raw data supporting the conclusions of this article will be made available by the authors, without undue reservation.

## Ethics statement

The studies involving human participants were reviewed and approved by the Ethics Committee of College of Philosophy and Sociology of Jilin University. Written informed consent to participate in this study was provided by the participants’ legal guardian/next of kin.

## Author contributions

LC conceived the study and designed the trial and drafted the manuscript. ZL and LC supervised the conduct of the trial and data collection, provided statistical advice on study design, and analyzed the data. ZL took responsibility for the manuscript as a whole. All authors contributed substantially to its revision.

## Conflict of interest

The authors declare that the research was conducted in the absence of any commercial or financial relationships that could be construed as a potential conflict of interest.

## Publisher’s note

All claims expressed in this article are solely those of the authors and do not necessarily represent those of their affiliated organizations, or those of the publisher, the editors and the reviewers. Any product that may be evaluated in this article, or claim that may be made by its manufacturer, is not guaranteed or endorsed by the publisher.
